# Comparative Performance of Body Composition Parameters in Prediction of Death in Hospitalized Patients on Maintenance Hemodialysis: A Cohort Study

**DOI:** 10.1038/s41598-020-67019-0

**Published:** 2020-06-23

**Authors:** Buyun Wu, Chenyan Yan, Sufeng Zhang, Yifei Ge, Xueqiang Xu, Yajie Wang, Lin Xu, Chengning Zhang, Zhimin Huang, Haibin Ren, Jingjing Wu, Changying Xing, Huijuan Mao

**Affiliations:** 1 Department of Nephrology, The First Affiliated Hospital of Nanjing Medical University, Nanjing, 210029 China; 2 Department of Nephrology, The Affiliated Jiangning Hospital of Nanjing Medical University, Nanjing, 211100 China

**Keywords:** Outcomes research, End-stage renal disease, Nutrition, Acid, base, fluid, electrolyte disorders, Nephrology, Risk factors

## Abstract

We compared the prognostic value of nutritional or volumetric parameters measured by body composition in hospitalized patients on maintenance hemodialysis. We conducted a cohort study to assess the association of different parameters of body composition with all-cause mortality in inpatients admitted to our nephrology department from January 2014 to December 2016. Of the 704 study patients, 160 (22.7%) died during a median follow-up of 33 months. In multivariate adjusted Cox models, higher ratio of extracellular water to body cell mass (ECW/BCM) (adjusted HR per 1-SD, 1.49; 95% CI, 1.19 to 1.85), lower lean tissue index (LTI) (adjusted HR per 1-SD, 0.70; 95% CI, 0.57 to 0.86) and lower body cell mass index (BCMI) (adjusted HR per 1-SD, 0.70; 95% CI, 0.58 to 0.85) were associated with a significantly greater risk of death. When these parameters were added to the fully adjusted model, BCMI performed best in improving the predictability for all-cause mortality (integrated discrimination improvement = 0.02, P = 0.04; net reclassification index = 0.11, P = 0.04). Among body composition indexes, ECW/BCM was the most relevant fluid volume indices to mortality and BCMI and LTI were the most relevant nutritional status indices to mortality in maintenance hemodialysis patients.

## Introduction

End-stage renal disease (ESRD) has become one of the major health problems in the world. In 2010, it was estimated that 284 individuals per million population were undergoing maintenance dialysis throughout the world^1^, and the number of dialysis patients is growing at an alarming rate^2–5^.

Dialysis is the major treatment for ESRD and brings a heavy economic burden to all countries^6^. Despite this, dialysis patients still have poor prognosis^5,7^. The main risk factors for the mortality includes vascular access, cardiovascular complications, cerebrovascular complications, infection, anemia, mineral metabolic disorders, renal osteopathy, fluid overload (FO) and malnutrition^7,8^.

Bioelectrical impedance analysis-based body composition analysis is widely used and accepted as an ideal tool for assessing fluid volume and nutritional status in maintenance dialysis patients^9^. It is easy-to-use, safe, noninvasive, repeatable and comprehensive. It has both anthropometric parameters in assessing fluid volume [such as overhydration (OH)^10^, OH/extracellular water (OH/ECW)^8,11–14^, the ratio of extracellular water to intracellular water (ECW/ICW)^15^, the ratio of extracellular water/body cell mass (ECW/BCM)^16,17^] and nutrition status [such as lean tissue index (LTI)^11,18–23^, fat tissue index (FTI)^11,18^, body cell mass index (BCMI)^24,25^, BCM/weight^26^]. However, it is still unclear which nutritional or volumetric parameters of body composition, measured by bioelectrical impedance, have the greatest prognostic value in maintenance hemodialysis patients.

We designed this cohort study to assess and compare the association of different parameters in body composition with all-cause medium-term mortality in hemodialysis patients.

## Results

### Baseline data of the study population

**Figure 1:**
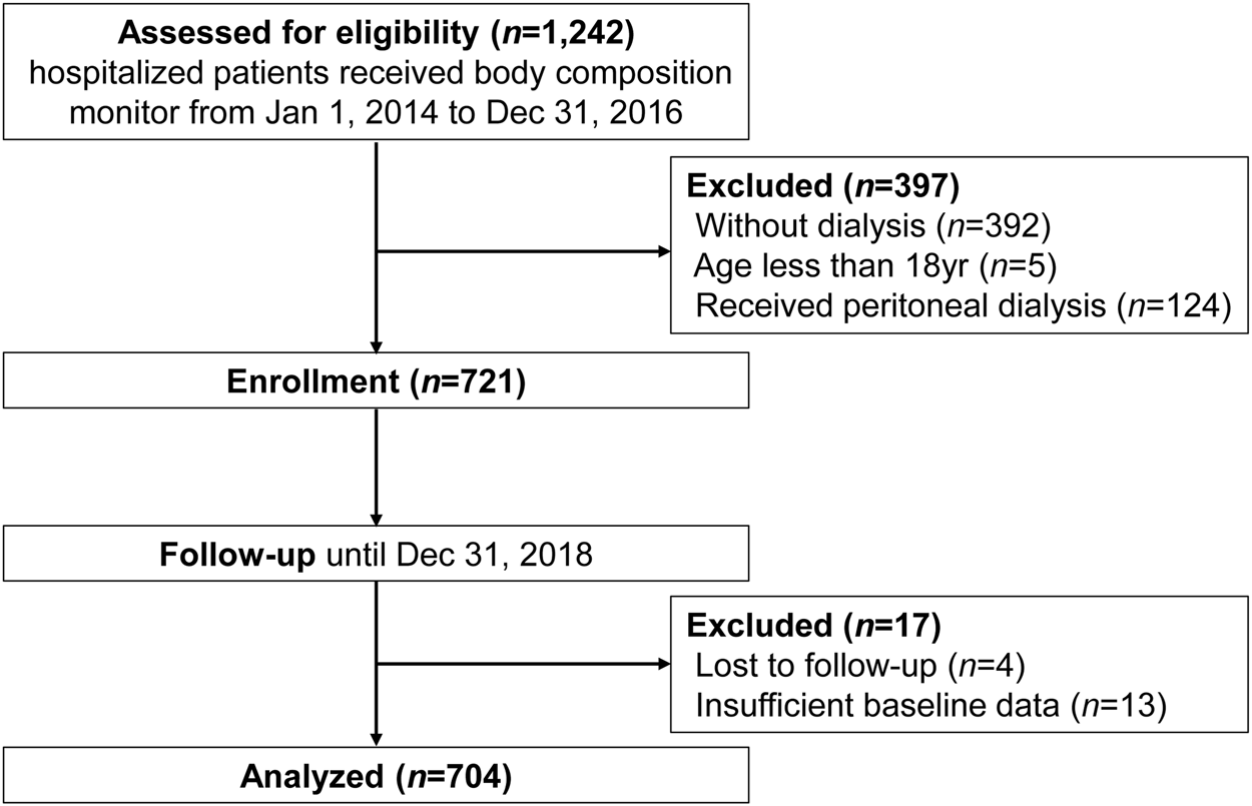
Study design flow chart.

We screened 1242 inpatients receiving BCM screening, of whom 721 were on maintenance hemodialysis and were enrolled in this study. After excluding 4 patients who were lost to follow-up and 13 had insufficient baseline data, 704 patients were finally analyzed (Fig. 1).

The study population included 406 males and 298 females. The mean age was 54 ± 15 years, and the median dialysis vintage was 47 (1–92) months. The median follow-up time was 33 (26–37) months. During the follow-up period, 160 (22.7%) patients died. The 1-year mortality and 2-year mortality were 8.9% and 16.4%, respectively. Additional demographic, clinical, and laboratory parameters were shown in Table 1.

**Table 1:** Baseline characteristics of study participants at the time of study enrollment.

Variables	All (N = 704)	Survivors (N = 544)	Non-survivors (N = 160)	P value
**Demographic data**				
Age (years)	54 ± 15	51 ± 14	64 ± 15	<0.001
Sex (male: female)	406:298	321:223	85:75	0.19
Height (cm)	165.2 ± 7.6	165.6 ± 7.6	164.1 ± 7.7	0.08
Weight (Kg)	61.2 ± 12.4	61.7 ± 12.4	59.0 ± 11.7	0.01
Body mass index (Kg/m^2^)	22.3 ± 3.8	22.5 ± 3.7	21.8 ± 3.9	0.04
Systolic blood pressure (mmHg)	139 ± 21	139 ± 20	140 ± 24	0.38
Diastolic blood pressure (mmHg)	82 ± 13	83 ± 13	78 ± 13	<0.001
Mean arterial pressure (mmHg)	101 ± 14	102 ± 14	98 ± 15	0.08
**Primary disease (n, %)**				<0.001
Diabetic nephropathy	106(15)	59(11)	47(29)	
Others	598(85)	485(89)	113(71)	
**Comorbidity (n, %)**				
Hypertension	567(80)	436(80)	131(82)	0.63
Diabetes	165(23)	95(17)	70(44)	<0.001
Infection	189(27)	123(23)	66(41)	<0.001
Charlson comorbidity index	3.0 ± 1.3	2.8 ± 1.2	3.8 ± 1.4	<0.001
**Reasons for admission (n, %)**				
Vascular access	208(30)	156(29)	52(32)	0.35
Infection	90(13)	50(9)	40(25)	<0.001
Cardiovascular diseases	51(7)	32(6)	19(12)	0.01
Parathyroidectomy	265(38)	247(45)	18(11)	<0.001
Others	90(13)	59(11)	31(19)	0.005
**Dialysis data**				
Incident dialysis (%)	204(29.0)	153(28.1)	51(31.9)	0.36
Dialysis vintage (months)	47(1,92)	55(1,100)	22(1,66)	0.006
Using arteriovenous fistula (%)	439 (62)	350(64)	89(56)	0.05
Using deep vein catheter (%)	265(38)	194(36)	71(44)	0.05
**Laboratory data**				
Hemoglobin (g/L)	94.7 ± 23.5	95.5 ± 24.1	91.8 ± 21.3	0.09
Albumin (g/L)	35.3 ± 6.0	36.0 ± 5.8	33.0 ± 6.0	<0.001
Total cholesterol (mmol/L)	4.3 ± 1.3	4.3 ± 1.2	4.3 ± 1.4	0.65
Total triglycerides (mmol/L)	1.8 ± 1.5	1.7 ± 1.4	2.0 ± 1.6	0.09
HDL-C (mmol/L)	1.0 ± 0.3	1.0 ± 0.3	0.9 ± 0.3	0.001
LDL-C (mmol/L)	2.8 ± 0.9	2.8 ± 0.9	2.8 ± 1.0	0.94
Adjusted calcium (mmol/L)	2.4 ± 0.3	2.4 ± 0.3	2.4 ± 0.2	0.004
Phosphorus (mmol/L)	1.9 ± 0.6	1.9 ± 0.6	1.6 ± 0.6	<0.001
Intact parathyroid hormone (pg/mL)	421(156,1344)	601(200,1551)	223(96,429)	<0.001

**Abbreviations**: HDL-C, high-density lipoprotein cholesterol; LDL-C, low-density lipoprotein cholesterol.

### Anthropometric parameters of fluid volume and nutritional status

Body composition parameters were compared between two groups divided by survival status during the follow-up period (Table 2). The anthropometric parameters of fluid volume including OH, OH/ECW, FO%, ECW/BCM and ECW/ICW were 2.2 ± 2.5 L, 0.13 ± 0.12, 28%, 0.97 ± 0.32 L/Kg and 0.97 ± 0.17, respectively. The survivors had significantly lower OH, lower OH/ECW, lower ECW/BCM and lower ECW/ICW as compared to non-survivors.

**Table 2:** Body composition parameters in study participants.

Variables	All (N = 704)	Survivors (N = 544)	Non-survivors (N = 160)	P value
OH [L]	2.2 ± 2.5	2.1 ± 2.6	2.4 ± 2.2	0.004
OH/ECW	0.13 ± 0.12	0.12 ± 0.13	0.15 ± 0.11	<0.001
FO (%)	196(28)	143(26)	53(33)	0.09
ECW/BCM (L/Kg)	0.97 ± 0.32	0.91 ± 0.26	1.16 ± 0.42	<0.001
ECW/ICW	0.97 ± 0.17	0.95 ± 0.17	1.05 ± 0.14	<0.001
FTI (Kg/m^2^)	9.8 ± 4.2	9.6 ± 4.0	10.7 ± 4.8	0.01
ATM/weight	0.43 ± 0.14	0.42 ± 0.13	0.47 ± 0.15	<0.001
LTI (Kg/m^2^)	11.6 ± 2.5	12.0 ± 2.3	10.1 ± 2.3	<0.001
LTM/weight	0.53 ± 0.13	0.55 ± 0.13	0.48 ± 0.14	<0.001
BCMI (Kg/m^2^)	6.10 ± 1.75	6.41 ± 1.66	5.03 ± 1.60	<0.001
BCM/weight	0.28 ± 0.09	0.29 ± 0.08	0.24 ± 0.08	<0.001

**Abbreviations**: ATM, adipose tissue mass; BCM, body cell mass; BCMI, body cell mass index; ECW, extracellular water; ECW/BCM, the ratio of extracellular water and body cell mass; ECW/ICW, the ratio of extracellular water and intracellular water; FO, fluid overload; FTI, fat tissue index; LTI, lean tissue index; LTM, lean tissue mass; OH, overhydration; OH/ECW, the ratio of overhydration and extracellular water.

The anthropometric parameters for nutritional status including body mass index (BMI), FTI, LTI and BCMI were 22.3 ± 3.8 Kg/m^2^, 9.8 ± 4.2 Kg/m^2^, 11.6 ± 2.5 Kg/m^2^ and 6.10 ± 1.75 Kg/m^2^, respectively (Table 1 and Table 2). The survivors had significantly lower FTI, higher LTI and higher BCMI as compared to non-survivors.

### Correlation between fluid volume parameters and nutritional parameters

The relationship between fluid volume and nutritional status were shown in scatterplots (Supplementary Fig. S1). In brief, ECW/BCM had a strong relationship with LTI (Spearman’s r = −0.78, P < 0.001) and BCMI (Spearman’s r = −0.79, P < 0.001). And there was a very good linear relationship (Spearman’s r = 0.99, P < 0.001) between LTI and BCMI (Supplementary Table S1). We further calculated the ratio of LTI to BCMI (LTI/BCMI) and the scatterplot with fluid volume parameters (Supplementary Fig. S2). We found that LTI/BCMI was strongly correlated with ECW/BCM (Spearman’s r = 0.79, P < 0.001) and ECW/ICW (Spearman’s r = 0.53, P < 0.001), weakly correlated with OH/ECW (Spearman’s r = 0.10, P = 0.008) and not correlated with OH (Spearman’s r = 0.03, P = 0.356).

### Univariate and multivariate analyses of measured indices and mortality

**Figure 2:**
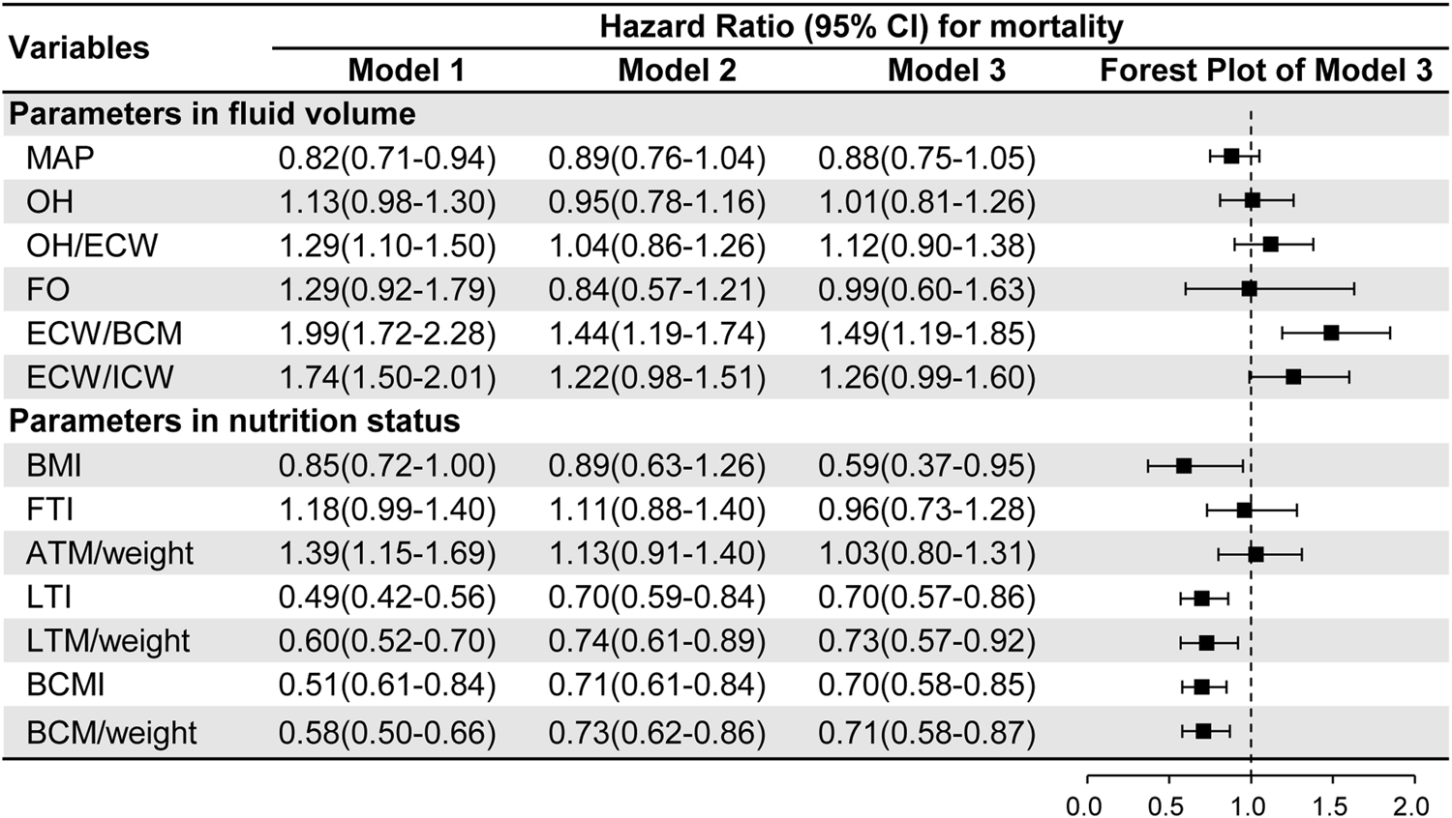
Multivariate Cox regression analysis of measured parameters in fluid volume and nutritional status for all-cause mortality (n = 704). **Abbreviations**: ATM, adipose tissue mass; BCM, body cell mass; BCMI, body cell mass index; BMI, body mass index; ECW, extracellular water; ECW/BCM, the ratio of extracellular water and body cell mass; ECW/ICW, the ratio of extracellular water and intracellular water; FO, fluid overload; FTI, fat tissue index; LTI, lean tissue index; LTM, lean tissue mass; MAP, mean arterial pressure; OH, overhydration; OH/ECW, the ratio of overhydration and extracellular water. **Note**: All parameters were natural log transformed (except OH, OH/ECW and FO) and standardized to 1 SD. Model 1: univariate Cox regression. Model 2: model 1+ age, weight, diabetes, modified Charlson comorbidity index, serum albumin concentrations, HDL-C, incident dialysis and admission due to parathyroidectomy. Model 3: model 2 + sex, smoking status, hypertension, mean arterial pressure, dialysis vintage, using DVC, hemoglobin, total triglycerides, adjusted calcium, phosphorus and iPTH and admission due to other reasons (including vascular access, infection, cardiovascular disease and others).

We assessed the measured indices using three models (Fig. 2): model 1 was unadjusted; model 2 was adjusted with age, weight, diabetes, modified Charlson comorbidity index (CCI), serum albumin, high-density lipoprotein cholesterol (HDL-C), incident hemodialysis and admission due to parathyroidectomy, which were identified using stepwise Cox regression to predict prognosis (Supplementary Tables S2–3); model 3 was further adjusted with other variables based on model 2, including sex, smoking status, hypertension, mean arterial pressure (MAP), dialysis vintage, using deep vein catheter (DVC), hemoglobin, total triglycerides, adjusted calcium, phosphorus and intact parathyroid hormone (iPTH) and admission due to other reasons (including vascular access, infection, cardiovascular disease and others).

Model 1 showed that higher fluid volume indices (OH, OH/ECW, ECW/BCM and ECW/ICW), higher body fat ratio [FTI and adipose tissue mass (ATM)/weight], lower lean tissue ratio indices [LTI, lean tissue mass (LTM)/weight], and lower BCM ratio (BCMI and BCM/weight) were all associated with higher mortality during follow-up.

In fully adjusted analyses (model 3), ECW/BCM, LTI, BCMI and BCM/weight were associated with death (Fig. 2). Higher ECW/BCM and lower LTI were both associated with higher mortality (adjusted hazard ratio (HR) per 1-SD higher ln[ECW/BCM], 1.49; 95% confidence interval [95% CI], 1.19 to 1.85; adjusted HR per 1-SD higher ln[LTI], 0.70; 95% CI, 0.57 to 0.86). Like LTI, lower BCMI was associated with higher mortality (adjusted HR per 1-SD higher ln[BCMI], 0.70; 95% CI, 0.58 to 0.85). The higher LTM/weight and BCM/weight were also associated with reduced risk of death (Fig. 2).

### Correlation analysis of quartiles of indicators with mortality

**Figure 3:**
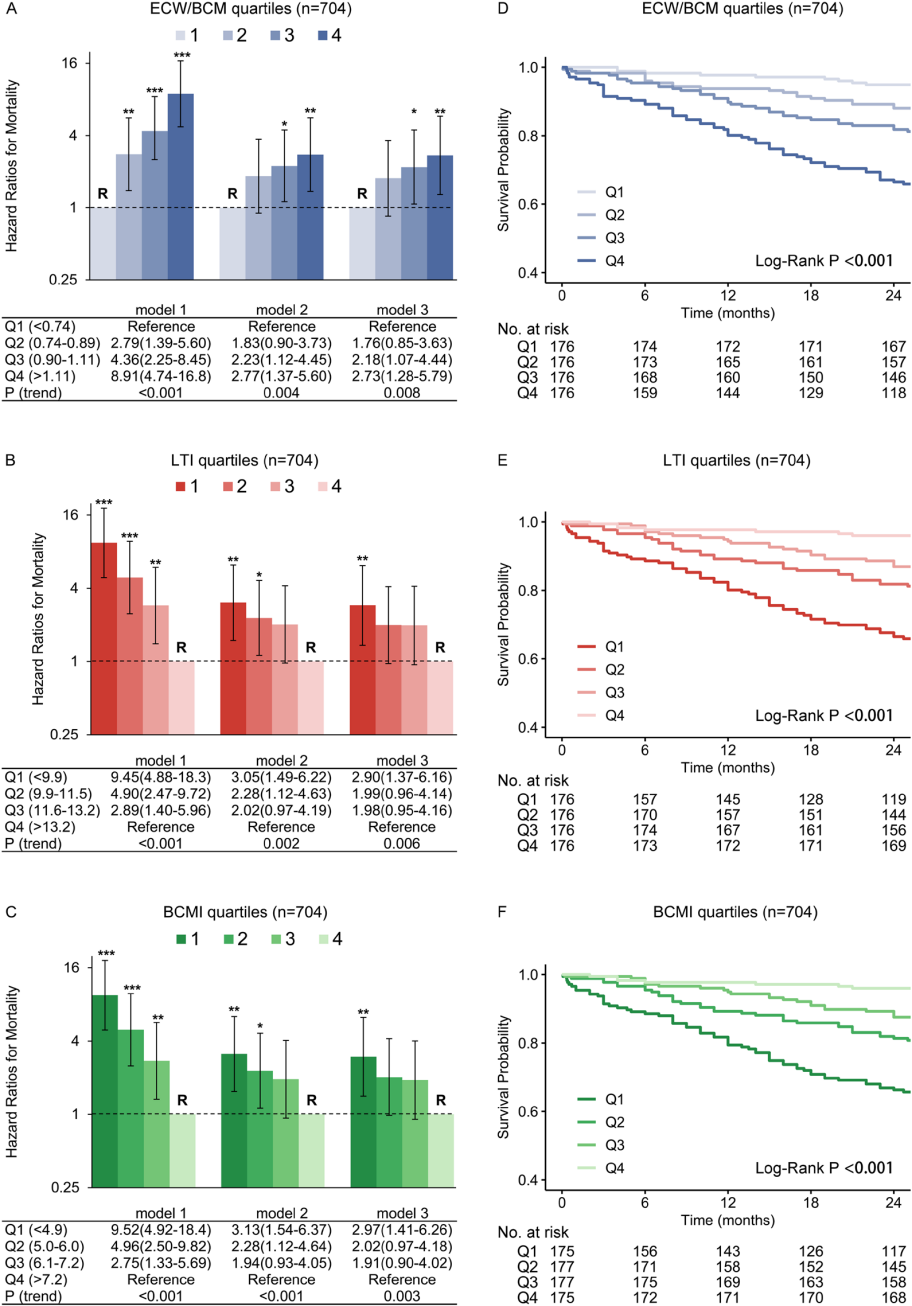
Hazard ratios and survival curves of 4 groups classified by quartiles of measured parameters in fluid volume (ECW/BCM) and nutritional status (LTI and BCMI). **Abbreviations**: ECW/BCM, the ratio of extracellular water and body cell mass; LTI, lean tissue index. **Note**: (**A–C**) Showed the hazard ratios and 95% confidence intervals for all-cause mortality according to quartiles of ECW/BCM, LTI and BCMI, respectively. Model 1 was unadjusted; model 2 was adjusted for age, weight, diabetes, modified Charlson comorbidity index, serum albumin concentrations, HDL-C, incident dialysis and admission due to parathyroidectomy; and model 3 incorporated the aforementioned variables in addition to sex, smoking status, hypertension, mean arterial pressure, dialysis vintage, using DVC, hemoglobin, total triglycerides, adjusted calcium, phosphorus and iPTH and admission due to other reasons (including vascular access, infection, cardiovascular disease and others). Higher quartiles of ECW/BCM were associated with monotonic increase in the risk of death in unadjusted and adjusted analyses (**A**). In contrast, lower quartiles of LTI (**B**) and BCMI (**C**) were associated with monotonic increase in the risk of death in unadjusted and adjusted analyses. We found similar results for quartiles of ECW/BCM, LTI and BCMI using time-to-event analyses in the first 24 months (**D–F**).

Indicators of ECW/BCM, LTI and BCMI were assessed by quartiles. We found that higher quartiles of ECW/BCM were associated with an increased risk of death in the unadjusted analyses (Fig. 3A). In fully adjusted analyses (model 3), patients with ECW/BCM in the highest quartiles compared with the lowest quartiles had a 2.73 greater HR for death (95% CI, 1.28 to 5.79). Similarly, we found that lower quartiles of LTI and BCMI were associated with an increased risk of death in the unadjusted analyses (Fig. 3B,C). In the fully adjusted analyses (model 3), patients with LTI or BCMI in the lowest quartile compared with the highest quartile had a 2.90 greater HR for death (95% CI, 1.37 to 6.16) or a 2.97 greater HR of death (95% CI, 1.41 to 6.26), respectively. We found similar results in quartiles of ECW/BCM, LTI and BCMI using time-to-event analyses in the first 24 months (Fig. 3D–F). Lowest quartiles of LTM/weight and BCM/weight did not associate with mortality in the full adjusted model (Supplementary Fig. S3).

The adjusted restricted cubic spline models revealed that there was a linear relationship between ECW/BCM, LTI, BCMI and all-cause mortality (Supplementary Tables 4–5) after adjusted all the variables. In contrast, a U-shape relationship between FTI or ATM/weight and all-cause mortality may exist, although patients with FTI or ATM/weight higher than 95% percentile did not have significantly increased risk of mortality as compared to those with median FTI or ATM/weight.

### Comparison of predictive value of indictors for mortality

To clarify the additive predictive power of indictors for mortality, we calculated the NRI and the IDI (Table 3). When LTI was added to the base model, it showed a significant improvement in predicting all-cause mortality compared with the basic model (IDI = 0.01, 95% CI 0.00 to 0.04, P = 0.04; NRI = 0.11, 95% CI 0.01 to 0.21, P = 0.03). BCMI had a weaker effect as compared to LTI (IDI = 0.02, 95% CI 0.01 to 0.04, P = 0.05; NRI = 0.10, 95% CI −0.00 to 0.21, P = 0.09). In the fully adjusted model, the improvement due to adding of BCMI was significant (IDI = 0.02, 95% CI 0.00 to 0.04, P = 0.04; NRI = 0.11, 95% CI 0.00 to 0.23, P = 0.04). However, adding BMI, ECW/BCM, LTM/weight or BCM/weight to the basic model or full adjusted model did not improve the discriminative ability in each model for all-cause mortality.

**Table 3:** Predictability of Cox regression models for all-cause mortality using net reclassification index, integrated discrimination improvement, and C-statistic.

Models	IDI (95% CI)	*P* Value	NRI (95% CI)	*P* Value	C-statistics (95% CI)	Mean of Difference in C-statistics (95% CI)^a^
Basic Model A^b^	reference		reference		0.80(0.76,0.83)	
Model A + BMI	0.00(−0.00,0.02)	0.58	−0.01(−0.16,0.17)	0.75	0.80(0.76,0.83)	0.00(0.00,0.00)
Model A + ECW/BCM	0.01(0.00,0.04)	0.07	0.12(−0.00,0.21)	0.05	0.81(0.76,0.83)	0.01(0.00,0.03)
Model A + LTI	0.01(0.00,0.04)	0.04	0.11(0.01,0.21)	0.03	0.81(0.78,0.84)	0.01(0.00,0.03)
Model A + BCMI	0.02(0.01,0.04)	0.05	0.10(−0.00,0.21)	0.09	0.81(0.78,0.84)	0.01(0.00,0.03)
Model A + LTM/weight	0.01(−0.00,0.04)	0.21	0.07(−0.12,0.19)	0.21	0.80(0.77,0.83)	0.01(0.00,0.02)
Model A + BCM/weight	0.01(−0.00,0.04)	0.10	0.09(−0.03,0.20)	0.15	0.80(0.77,0.84)	0.01(0.00,0.02)
Full adjusted Model B^c^	reference		reference		0.81(0.78,0.84)	
Model B + BMI	0.01(−0.00,0.04)	0.18	0.10(−0.14,0.22)	0.18	0.81(0.78,0.84)	0.00(−0.00,0.01)
Model B + ECW/BCM	0.01(−0.00,0.04)	0.08	0.15(−0.01,0.25)	0.07	0.81(0.78,0.84)	0.00(−0.00,0.01)
Model B + LTI	0.01(−0.00,0.04)	0.07	0.11(−0.02,0.23)	0.09	0.82(0.79,0.84)	0.01(0.00,0.02)
Model B + BCMI	0.02(0.00,0.04)	0.04	0.11(0.00,0.23)	0.04	0.82(0.79,0.85)	0.01(0.00,0.02)
Model B + LTM/weight	0.01(−0.00,0.03)	0.22	0.09(−0.06,0.19)	0.21	0.81(0.78,0.84)	0.00(−0.00,0.02)
Model B + BCM/weight	0.01(−0.00,0.04)	0.10	0.09(−0.05,0.23)	0.13	0.81(0.78,0.84)	0.00(−0.00,0.02)

**Abbreviations**: 95% CI, 95% confidence interval; BCM, body cell mass; BCMI, body cell mass index; BMI, body mass index; ECW/BCM, the ratio of extracellular water and body cell mass; IDI, integrated discrimination improvement; LTI, lean tissue index; LTM, lean tissue mass; NRI, net reclassification index.

**Note:** ^a^Differences in C-statistics were calculated using bootstrapping with 1000 replicates.

^b^Basic Model A included age, weight, diabetes, modified Charlson comorbidity index, serum albumin concentrations, HDL-C, incident dialysis and admission due to receiving parathyroidectomy. The t0 was set at 24 months in R 3.6.

^c^Full adjusted Model B included model A and sex, smoking status, hypertension, mean arterial pressure, dialysis vintage, using DVC, hemoglobin, total triglycerides, adjusted calcium, phosphorus and iPTH and other admission reasons. The t0 was set at 24 months in R 3.6.

In addition, the C-statistics of the basic model with LTI significantly increased compared with the basic model (mean difference in C-statistics = 0.01; 95% CI, 0.00 to 0.03) or with the fully adjusted model (mean difference in C-statistics = 0.01; 95% CI, 0.00 to 0.02). BCMI had similar discriminative ability with LTI. Despite of small IDI, NRI and C-statistics, the results proved that LTI or BCMI improve the classification of predicting 2-year mortality.

ROC curve analysis (Supplementary Fig. S4) indicated that the optimal ECW/BCM ratio cut-off point for predicting 2-year mortality was 1.07 L/Kg (sensitivity: 55.2%; specificity: 76.2%) which yielded a AUC of 0.71 (95% CI: 0.68 to 0.74). The similar effects were for LTI (cut-off point 10.8 Kg/m^2^, sensitivity: 66.4%; specificity: 67.2%) and BCMI (cut-off point 6.13 Kg/m^2^, sensitivity: 79.3%; specificity: 53.7%), which yielded almost the same AUC values.

### Subgroup analysis

**Figure 4:**
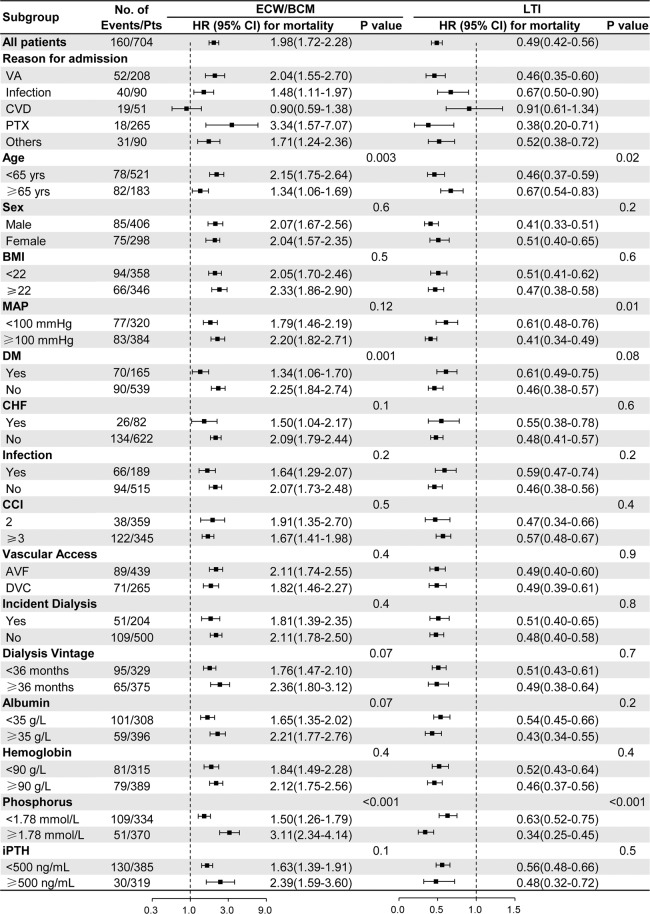
Subgroup analyses of the relationship between ECW/BCM or LTI and mortality in different groups. **Abbreviations**: AVF, arteriovenous fistula as vascular access; BMI, body mass index; CCI, Charlson comorbidity index; CHF, congestive heart failure; CVD, cardiovascular disease; DM, diabetes mellitus; DVC, deep venous catheter; ECW/BCM, the ratio of extracellular water and body cell mass; iPTH, intact parathyroid hormone; LTI, lean tissue index; MAP, mean arterial pressure; PTX, parathyroidectomy; VA, vascular access. **Note**: ECW/BCM and LTI associate with death across subgroups. Unadjusted Hazard ratios for mortality according to ECW/BCM and LTI across subgroups were showed. ECW/BCM and LTI were natural log transformed and standardized to 1 SD. P values refer to the significance of interaction terms testing for effect modification by subgroup.

Correlation of ECW/BCM or LTI to the risk of death were analyzed in the subgroups (Fig. 4) based on sex, BMI, MAP, diabetes, chronic heart failure, infection, CCI, incident dialysis, hemodialysis vintage, vascular access, serum albumin, hemoglobin and iPTH. Significant differences were observed in subgroup analyses based on the age, MAP, diabetes mellitus and phosphorus (some p < 0.05). The association of ECW/BCM with mortality became weak in patients with age more than 65 years, with diabetes, with serum phosphorus less than 1.78 mmol/L. Also, the association of LTI with mortality became weak in patients with age more than 65 years, with lower MAP, or with serum phosphorus less than 1.78 mmol/L. The subgroup analyses for BCMI were similar with LTI (Supplementary Fig. S5). Specifically, the association between ECW/BCM or LTI and mortality became insignificant in patients those admitted for cardiovascular disease.

## Discussion

Measuring parameters in body composition are essential to high quality care in dialysis patients and need further study. To the best of our knowledge, this is the first article to compare the different anthropometric parameters from body composition analysis and prognosis evaluation. In this study, we found that the ECW/BCM of fluid volume indices and LTI and BCMI of nutritional status indices were most relevant to mortality. Moreover, we calculated the cut-off values of ECW/BCM, LTI and BCMI in hemodialysis patients, which would be a reference for the intervention in fluid volume and nutritional status in future.

FO was independent with adverse prognosis in dialysis patients. Previous studies reported OH^10^, OH/ECW^8,11–14,27^, ECW/BCM^17^, ECW/ICW^15^ were associated with mortality. It was still unclear which fluid parameters in body composition were most associated with mortality. Overhydration (OH) reflects overhydration in absolute liters through a mathematic model^28^ and OH/ECW reflects relative overhydration in percent compared to ECW. Our study showed that OH and OH/ECW in univariate analyses were significantly associated with mortality in hemodialysis inpatients, but the effect disappeared after adjusting for other variables. This may be explained by the OH, OH/ECW and ECW/ICW value in hospitalized patients being confounded by other variables such as infection, the Charlson comorbidity index and intervention by physicians. Moreover, OH, OH/ECW or ECW/ICW at a single time point cannot reflect the long-term fluid overload, which has a higher risk of death than that solely on the single measurement^8^. In contrast, our study showed that ECW/BCM were independently associated with all-cause mortality. This finding may be because ECW/BCM is a hybrid index of wasting and fluid overload^16^, which strongly correlated with ECW/ICW, LTI or BCMI in this study. Moreover, we suggested ECW/BCM should be controlled below 1.07 Kg/m^2^, lower than 1.20 Kg/m^2^ proposed by Ruperto *et al*.^16^. These results suggested that the physician should pay more attention to ECW/BCM, and be actively involved in optimizing the hydrational status of inpatients using bioelectrical impedance^27,29^.

Anthropometric parameters in nutrition included LTI, LTM/weight, FTI, ATM/weight, BCMI and BCM/weight in body composition. The lean tissue is composed of muscles, organs, blood and bones and can estimate the muscle mass of the whole body to reflect the storage of proteins. Hemodialysis patients presented with a decrease in LTI^30^, which was associated with adverse prognosis^11,18–23^. Our results proved that the LTM and BCM standardized by height square (LTI and BCMI) were more associated with mortality compared with those standardized by weight (LTM/weight and BCM/weight), which may be influenced by the hydration status that was very common in hemodialysis patients. Extreme high LTM/weight or BCM/weight also meant relatively low ATM/weight, and vice versa. This was in accordance with a large international study which indicated the best survival in patients with both LTI and FTI in the 10th–90th percentiles of a healthy population^18^. Our study also confirmed that LTI was associated with mortality more robustly than FTI, because higher LTI presented as the more storage of proteins and energy reserve which involved in metabolic process. Therefore, a physician should take into consideration intradialytic parenteral nutrition therapy^31^, correcting metabolic acidosis^32^, and encouraging excise^33,34^ to reduce muscle wasting^35^.

BCM is defined as lean body mass without bone mineral mass or extracellular water, and is the most metabolically active body compartment^26^. Thus, it would not be influenced by overhydration and independently correlated with mortality in dialysis patients^11,24,25^. Our research confirmed that BCMI had a good liner relationship with LTI, and LTI/BCMI strongly correlated with ECW/ICW instead of OH or OH/ECW. Also, the magnitude of the association between BCMI and mortality was similar with that between LTI and mortality. Meanwhile, the survival curves grouped by quartiles of BCMI were similar to LTI, and the additive predictive power of LTI and BCMI were also similar for mortality. These results that BCMI was not superior to LTI may be explained by the model for body composition had already discriminated for excess water from lean tissue mass (LTM + ATM + OH = weight)^28^. In this case, the calculated LTM was only influenced by hydration through excess intracellular water which was little changed even in hemodialysis^36^. Therefore, we propose that BCMI is equivalent to LTI as anthropometric parameters in nutrition when assessing dialysis inpatients using this body composition monitor.

There are some limitations in this study. First, only one time point for hospitalized patients was measured and analyzed in our research. This was because the patients came from different regions in several provinces, repeated body composition analysis can be barely detected during the follow-up. Second, we could not analyze the association between the causes of death and the above parameters in body composition, because nearly 30% of the patients died with an unknown reason. Third, dialysis dose that was associated with outcomes for hemodialysis patients was not included in the adjustment. This was because dialysis dose measured in hospitalized patients could not represented a stable dose, and their short-term impact on the survival need be further studied^37,38^. More studies with larger samples are needed to confirm our results.

In conclusion, our study found that the ECW/BCM was the most relevant to mortality in fluid volume indices, and LTI and BCMI were the two most relevant to mortality in nutritional status indices. Higher LTI, higher BCMI or lower ECW/BCM were significantly associated with a lower risk of death and exhibited a stronger association with mortality than other body composition parameters in inpatients with maintenance hemodialysis. These findings suggest that determining LTI, BCMI or ECW/BCM may be beneficial to predicting patient survival in patients on hemodialysis.

## Methods

### Design and ethics

This is a bidirectional cohort study. All the study patients underwent body composition analysis during hospitalization. The patients were treated according to clinical routine and followed up for at least 24 months since they received body composition analysis.

The authors assert that all methods contributing to this work comply with the ethical standards of the relevant national and institutional committees on human experimentation and with the Helsinki Declaration 1975, as revised in 2008. The study was approved by the Ethics Committee of the First Affiliated Hospital of Nanjing Medical University (Jiangsu Province Hospital) (2017-SR-287). The Ethics Committee waived the need for informed consent as the detection of body composition was noninvasive and regularly used in this hospital and the data were analyzed anonymously.

### Study population

Adult maintenance hemodialysis patients admitted to the nephrology department of Jiangsu Province Hospital from January 2014 to December 2016, who were regularly assessed for body composition by bioelectrical impedance analysis within the first 3 days and survived more than 1 week after admission, were continuously enrolled in this study. The reasons for admission were unlimited. The patients received hemodialysis at least 3 times per week during hospitalization. Each duration, anticoagulant dosage and ultrafiltration rate per session of hemodialysis were formulated by renal physicians.

### Body composition analysis

Body composition was measured by a body composition monitor (Fresenius Medical Care, Bad Homburg, Germany) by the same experienced nurse in accordance with the instrument instructions on the morning after dialysis day. The monitor was validated against gold standard references (bromide and deuterium dilution) and showed excellent accordance^39^. The main parameters included height (m), weight (Kg), BMI (Kg/m^2^), ECW (L), ICW (L), OH (L), LTM (Kg), LTI (Kg/m^2^), ATM (Kg), FTI (Kg/m^2^), BCM (Kg) and BCMI (Kg/m^2^).

OH was determined by the body composition monitor in absolute liters independent of body composition by use of a physiological model based on normal tissue hydration^28^. Patients were considered to be FO when their OH/ECW was ≥15% in men and ≥13% in women^8^, which coincided with an absolute OH of about 2.5 L. LTI, FTI and BCMI were obtained by lean tissue mass, fat tissue mass and BCM divided by height in meters squared, respectively. BCM was defined as lean body mass without bone mineral mass or extracellular water, and was the most metabolically active body compartment^26^.

We grouped the above anthropometric parameters into two aspects: fluid volume and nutritional status. The former included MAP [as a reference due to its association with fluid volume^40^], OH, OH/ECW, FO, ECW/BCM and ECW/ICW. The latter included BMI (as a reference due to its association with nutritional status^41^), FTI, ATM/weight, LTI, LTM/weight, BCMI and BCM/weight.

### Data collection and outcome

The data collected included demographic data (name, age, sex, height, weight), clinical data [primary disease of chronic kidney disease, comorbidity, modified CCI (except the assessment of diabetes)], hemodialysis data (hemodialysis vintage, hemodialysis vascular access), reason for admission (including vascular access, infection, cardiovascular disease, parathyroidectomy and others), laboratory data [hemoglobin, albumin, total cholesterol, total triglycerides (TG), low-density lipoprotein cholesterol (LDL-C), HDL-C, serum adjusted calcium, serum phosphorus, iPTH] and the above parameters of body composition.

The primary outcome was all-cause mortality. All the patients were followed up for at least 24 months since receiving body composition analysis and had a definite result of 2-year all-cause mortality.

### Statistical methods

Statistical analyses were performed using SAS version 9.2 (SAS Institute, Cary, NC, USA) and R version 3.6 (R Foundation for Statistical Computing, Vienna, Austria; www.r-project.org). Continuous variables were expressed as the mean ± SD or the median (interquartile range [IQR]), and categorical variables were expressed as a number (percentage). We compared baseline characteristics and measured parameters in body composition between patients alive versus dead during follow-up using the Wilcoxon rank sum and chi-squared tests for continuous and categoric variables, respectively.

We used univariate (model 1) and multivariate (models 2 and 3) Cox regression to test the associations between measured parameters and mortality. In these models, measured parameters in body composition were natural log transformed (except OH and OH/ECW) and normalized to 1 SD to allow for comparison across parameters. Model 2 was adjusted for covariates according to multivariate Cox regression by stepwise selection [age, weight, diabetes, modified CCI, serum albumin concentrations, HDL-C, incident hemodialysis and admission due to receiving parathyroidectomy]. Model 3 was further adjusted by sex, smoking status, hypertension, MAP, dialysis vintage, using DVC, hemoglobin, total triglycerides, adjusted calcium, phosphorus and iPTH and admission due to other reasons. We also assessed potential nonlinear associations using restricted cubic spline models between continuous variables and outcomes^42^.

ECW/BCM, LTI, BCMI, LTM/weight and BCM/weight were divided into quartiles to test for nonlinear associations with mortality. We used Cox regression and adjusted for the same covariates as above. The associations between quartiles of the above five parameters with death were depicted using Kaplan-Meier curves, and the log rank test to compare rates of death across quartiles.

To test our hypothesis, that ECW/BCM, LTI or BCMI may be a better predictor of mortality than BMI, LTM/weight and BCM/weight, we assessed the additional effect of these parameters on two models. Model A was constructed including age, weight, diabetes, modified CCI, serum albumin concentrations, HDL-C, incident dialysis and admission due to receiving parathyroidectomy, which were obtained from a multivariate Cox regression with stepwise selection. Model B added other parameters [sex, smoking status, hypertension, mean arterial pressure, dialysis vintage, using DVC, hemoglobin, total triglycerides, adjusted calcium, phosphorus, iPTH and admission due to other reasons] in model A. Then, the integrated discrimination improvement (IDI) and the net reclassification index (NRI) were calculated to ascertain which body composition indices improved the discriminatory ability when added to the two models^43^. Furthermore, Harrell C index and differences in the C-statistics were also calculated using bootstrapping with 1000 replicates.

The receiving operating characteristic (ROC) curve was estimated using a nonparametric method to determine the cut-off value of the parameters for correctly identifying patients on the 2-year all-cause death.

For sensitivity, we performed subgroup analyses and assessed for effect modification according to baseline characteristics by testing the significance of interaction terms (subgroup × ECW/BCM or LTI). For all analyses, a two-tailed P value less than 0.05 was considered statistically significant.

## Supplementary information

Supplementary information.

## Data Availability

All data generated or analyzed during this study are included in this published article.

## References

[1] Thomas B (2015). Maintenance Dialysis throughout the World in Years 1990 and 2010. J. Am. Soc. Nephrol..

[2] Sun L (2016). Forecast of the incidence, prevalence and burden of end-stage renal disease in Nanjing, China to the Year 2025. BMC Nephrol..

[3] Bujang MA (2017). Forecasting the Incidence and Prevalence of Patients with End-Stage Renal Disease in Malaysia up to the Year 2040. Int. J. Nephrol..

[4] Tucker PS, Kingsley MI, Morton RH, Scanlan AT, Dalbo VJ (2014). The increasing financial impact of chronic kidney disease in australia. Int. J. Nephrol..

[5] Saran R (2019). US Renal Data System 2018 Annual Data Report: Epidemiology of Kidney Disease in the United States. Am. J. Kidney Dis..

[6] Karopadi AN, Mason G, Rettore E, Ronco C (2013). Cost of peritoneal dialysis and haemodialysis across the world. Nephrol. Dial. Transpl..

[7] Webster AC, Nagler EV, Morton RL, Masson P (2017). Chronic Kidney Disease. Lancet.

[8] Zoccali C (2017). Chronic Fluid Overload and Mortality in ESRD. J. Am. Soc. Nephrol..

[9] Erdogan E (2013). Reliability of bioelectrical impedance analysis in the evaluation of the nutritional status of hemodialysis patients - a comparison with Mini Nutritional. Assessment. Transpl. Proc..

[10] Mathew S (2015). Body composition monitoring and nutrition in maintenance hemodialysis and CAPD patients–a multicenter longitudinal study. Ren. Fail..

[11] Caetano C, Valente A, Oliveira T, Garagarza C (2016). Body Composition and Mortality Predictors in Hemodialysis Patients. J. Ren. Nutr..

[12] Kim YJ (2015). Overhydration measured by bioimpedance analysis and the survival of patients on maintenance hemodialysis: a single-center study. Kidney Res. Clin. Pract..

[13] Wizemann V (2009). The mortality risk of overhydration in haemodialysis patients. Nephrol. Dial. Transpl..

[14] Jotterand Drepper V (2016). Overhydration Is a Strong Predictor of Mortality in Peritoneal Dialysis Patients - Independently of Cardiac Failure. PLoS One.

[15] Chen W, Guo LJ, Wang T (2007). Extracellular water/intracellular water is a strong predictor of patient survival in incident peritoneal dialysis patients. Blood Purif..

[16] Ruperto, M., Sanchez-Muniz, F. J. & Barril, G. Extracellular mass to body cell mass ratio as a potential index of wasting and fluid overload in hemodialysis patients. A case-control study. *Clin Nutr*, 10.1016/j.clnu.2019.04.021 (2019). 10.1016/j.clnu.2019.04.021 31060893

[17] Avram, M. M., Fein, P. A., Borawski, C., Chattopadhyay, J. & Matza, B. Extracellular mass/body cell mass ratio is an independent predictor of survival in peritoneal dialysis patients. *Kidney Int Suppl*, S37-40, 10.1038/ki.2010.192 (2010). 10.1038/ki.2010.192 20671743

[18] Marcelli D (2015). Body composition and survival in dialysis patients: results from an international cohort study. Clin. J. Am. Soc. Nephrol..

[19] Rosenberger J, Kissova V, Majernikova M, Straussova Z, Boldizsar J (2014). Body composition monitor assessing malnutrition in the hemodialysis population independently predicts mortality. J. Ren. Nutr..

[20] Castellano S (2016). Risk identification in haemodialysis patients by appropriate body composition assessment. Nefrologia.

[21] Rymarz A, Gibinska J, Zajbt M, Piechota W, Niemczyk S (2018). Low lean tissue mass can be a predictor of one-year survival in hemodialysis patients. Ren. Fail..

[22] Zhang H, Tao X, Shi L, Jiang N, Yang Y (2019). Evaluation of body composition monitoring for assessment of nutritional status in hemodialysis patients. Ren. Fail..

[23] Parthasarathy, R., Oei, E. & Fan, S. L. Clinical value of body composition monitor to evaluate lean and fat tissue mass in peritoneal dialysis. *Eur J Clin Nutr*, 10.1038/s41430-019-0391-3 (2019). 10.1038/s41430-019-0391-3 30647437

[24] Rondanelli M (2018). Beyond Body Mass Index. Is the Body Cell Mass Index (BCMI) a useful prognostic factor to describe nutritional, inflammation and muscle mass status in hospitalized elderly?: Body Cell Mass Index links in elderly. Clin. Nutr..

[25] Valente, A., Caetano, C., Oliveira, T. & Garagarza, C. Evaluating haemodialysis patient’s nutritional status: Body mass index or body cell mass index? *Nephrology (Carlton)*, 10.1111/nep.13527 (2018). 10.1111/nep.13527 30414231

[26] Oliveira CM (2010). The phase angle and mass body cell as markers of nutritional status in hemodialysis patients. J. Ren. Nutr..

[27] Machek P, Jirka T, Moissl U, Chamney P, Wabel P (2010). Guided optimization of fluid status in haemodialysis patients. Nephrol. Dial. Transpl..

[28] Chamney PW (2007). A whole-body model to distinguish excess fluid from the hydration of major body tissues. Am. J. Clin. Nutr..

[29] Onofriescu M (2014). Bioimpedance-guided fluid management in maintenance hemodialysis: a pilot randomized controlled trial. Am. J. Kidney Dis..

[30] Marcelli D (2016). Longitudinal Changes in Body Composition in Patients After Initiation of Hemodialysis Therapy: Results From an International Cohort. J. Ren. Nutr..

[31] Marsen TA, Beer J, Mann H, German I-TG (2017). Intradialytic parenteral nutrition in maintenance hemodialysis patients suffering from protein-energy wasting. Results of a multicenter, open, prospective, randomized trial. Clin. Nutr..

[32] Vashistha T, Kalantar-Zadeh K, Molnar MZ, Torlen K, Mehrotra R (2013). Dialysis modality and correction of uremic metabolic acidosis: relationship with all-cause and cause-specific mortality. Clin. J. Am. Soc. Nephrol..

[33] Moraes C (2014). Resistance exercise: a strategy to attenuate inflammation and protein-energy wasting in hemodialysis patients?. Int. Urol. Nephrol..

[34] Kirkman DL (2014). Anabolic exercise in haemodialysis patients: a randomised controlled pilot study. J. Cachexia Sarcopenia Muscle.

[35] Ikizler TA (2013). Prevention and treatment of protein energy wasting in chronic kidney disease patients: a consensus statement by the International Society of Renal Nutrition and Metabolism. Kidney Int..

[36] El-Kateb S, Davenport A (2016). Changes in Intracellular Water Following Hemodialysis Treatment Lead to Changes in Estimates of Lean Tissue Using Bioimpedance. Spectroscopy. Nutr. Clin. Pract..

[37] Obialo CI, Hernandez B, Carter D (1998). Delivered dialysis dose is suboptimal in hospitalized patients. Am. J. Nephrol..

[38] Kanagasundaram NS (2012). Hemodialysis adequacy and the hospitalized end-stage renal disease patient–raising awareness. Semin. Dial..

[39] Wabel P, Chamney P, Moissl U, Jirka T (2009). Importance of whole-body bioimpedance spectroscopy for the management of fluid balance. Blood Purif..

[40] Sarafidis PA (2017). Hypertension in dialysis patients: a consensus document by the European Renal and Cardiovascular Medicine (EURECA-m) working group of the European Renal Association-European Dialysis and Transplant Association (ERA-EDTA) and the Hypertension and the Kidney working group of the European Society of Hypertension (ESH). Nephrol. Dial. Transpl..

[41] Carrero JJ, Avesani CM (2015). Pros and cons of body mass index as a nutritional and risk assessment tool in dialysis patients. Semin. Dial..

[42] Desquilbet L, Mariotti F (2010). Dose-response analyses using restricted cubic spline functions in public health research. Stat. Med..

[43] Pencina, M. J., D’Agostino, R. B. Sr., D’Agostino, R. B. Jr. & Vasan, R. S. Evaluating the added predictive ability of a new marker: from area under the ROC curve to reclassification and beyond. *Stat Med* **27**, 157–172; discussion 207–112, 10.1002/sim.2929 (2008). 10.1002/sim.2929 17569110

