# Supplementary material for: Comparative Performance of Body Composition Parameters in Prediction of Death in Hospitalized Patients on Maintenance Hemodialysis: A Cohort Study
Source: Sci Rep. 2020 Jun 23;10:10199. doi: 10.1038/s41598-020-67019-0 (PMC7311464; doi:10.1038/s41598-020-67019-0)
Supplement: Supplementary file 1 — Supplementary information. [file 41598_2020_67019_MOESM1_ESM.doc]

# Supplementary Materials

**Comparative Performance of Body Composition Parameters in Prediction of Death in Hospitalized Patients on Maintenance hemodialysis: A Cohort Study**

Buyun Wu, Chenyan Yan, Sufeng Zhang, Yifei Ge, Xueqiang Xu, Yajie Wang, Lin Xu, Chengning Zhang, Zhimin Huang, Haibin Ren, Jingjing Wu, Changying Xing, Huijuan Mao

**Outlines**

1. Figure S1. Scatterplot matrix for measured parameters.
2. Figure S2. Scatter plot of LTI/BCMI and measured parameters in fluid status.
3. Figure S3. Hazard ratios and survival curves of 4 groups classified by quartiles of LTM/weight and BCM/weight.
4. Figure S4. ROC curves for predicting 2-year mortality using ECW/BCM, LTI, BCMI, LTM/weight and BCM/weight.
5. Table S1. Matrix of Spearman correlation coefficients between measured parameters.
6. Table S2. Univariate COX regression models.
7. Table S3. Multivariate COX regression analysis of risk factors for death using traditional parameters except parameters in body composition.
8. Table S4. Summary of restricted cubic splines for the association between anthropometric parameters in fluid volume and mortality.
9. Table S5. Summary of restricted cubic splines for the association between anthropometric parameters in nutritional status and mortality.

**Figure S1. Scatterplot matrix for measured parameters.**


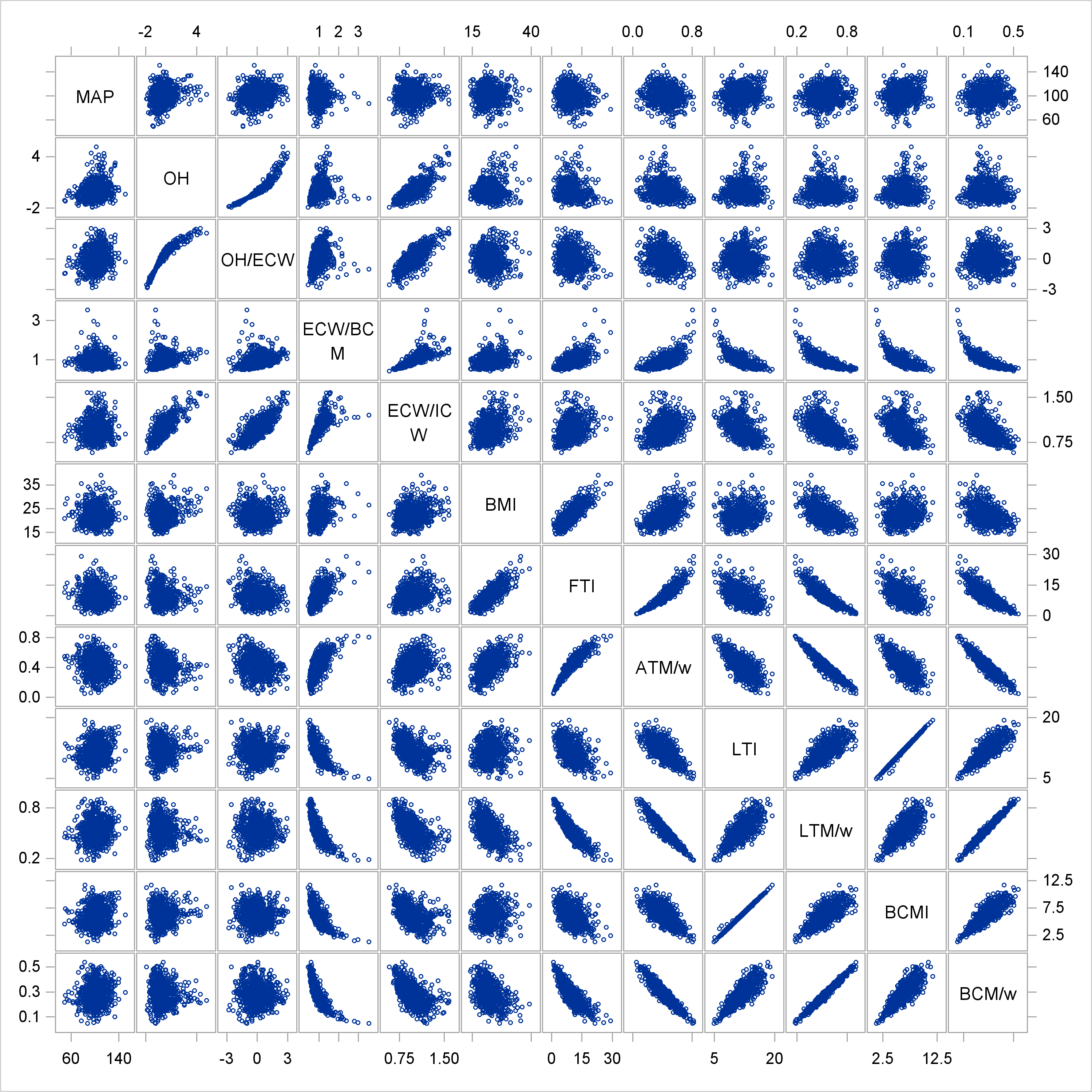


**Abbreviations:** ATM, adipose tissue mass; BCM, body cell mass; BCMI, body cell mass index; ECW, extracellular water; ECW/BCM, the ratio of extracellular water and body cell mass; ECW/ICW, the ratio of extracellular water and intracellular water; FTI, fat tissue index; LTI, lean tissue index; LTM, lean tissue mass; OH, overhydration; OH/ECW, the ratio of overhydration and extracellular water; w, weight.

**Figure S2. Scatter plot of LTI/BCMI and measured parameters in fluid status.**


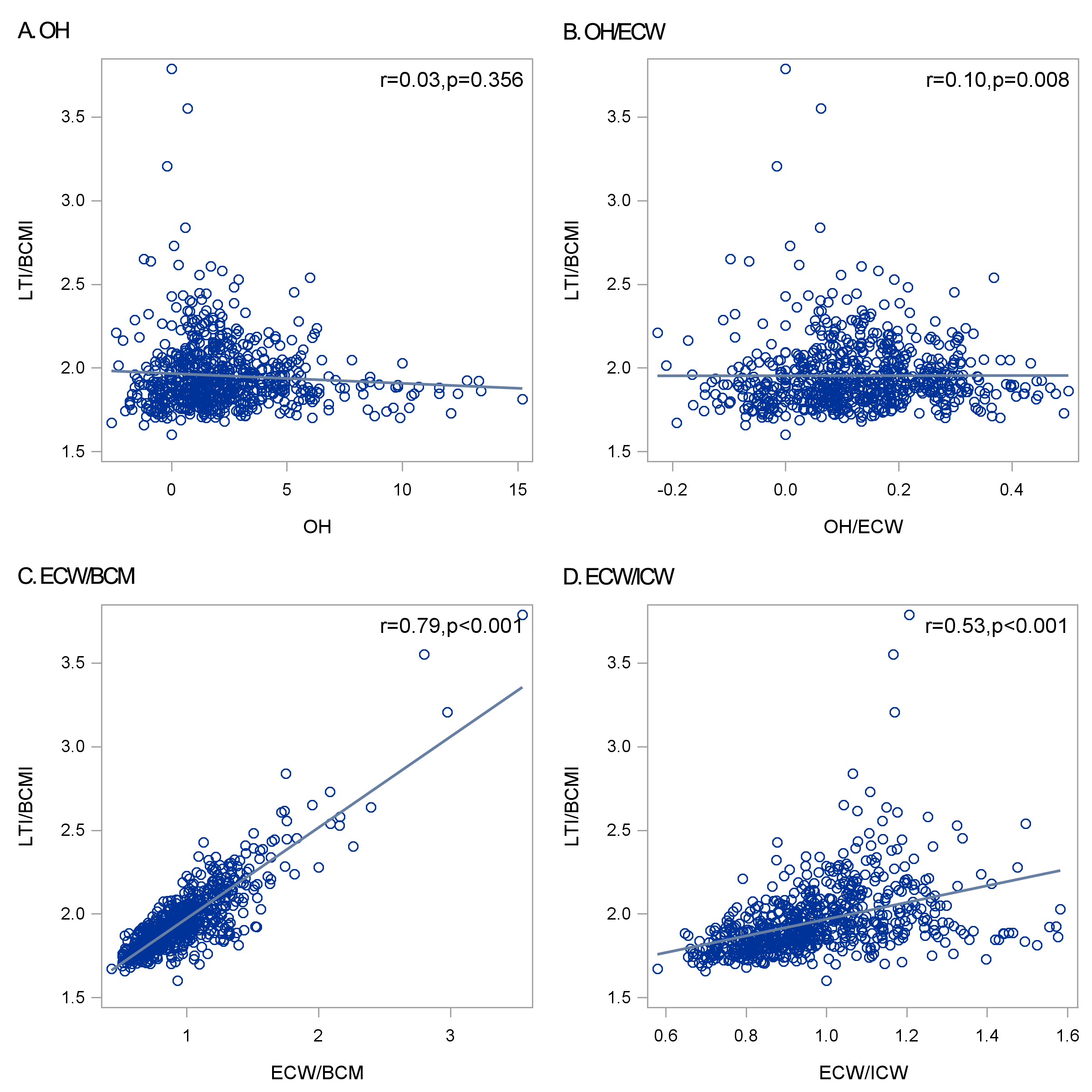


**Note:** The ratio of LTI and BCMI (LTI/BCMI) was slightly associated with OH/ECW, and strongly with ECW/BCM and ECW/ICW. Spearman correlation coefficients were calculated. **Abbreviations:** BCMI, body cell mass index; ECW, extracellular water; ECW/BCM, the ratio of extracellular water and body cell mass; ECW/ICW, the ratio of extracellular water and intracellular water; LTI, lean tissue index; OH, overhydration; OH/ECW, the ratio of overhydration and extracellular water.

**Figure S3. Hazard ratios and survival curves of 4 groups classified by quartiles of LTM/weight and BCM/weight.**





**Note:** Higher quartiles of LTM/weight were associated with monotonic increase in the risk of death in unadjusted analyses instead of adjusted analyses in Figure S3A. The similar association of BCM/weight and the risk of death were showed in Figure S3B. We found similar results for quartiles of LTM/weight and BCM/weight using time-to-event analyses in the first 24 months (Figure S3C-S3D).

**Abbreviations:** LTM, lean tissue mass; BCM, body cell mass.

**Figure S4. ROC curves for predicting 2-year mortality using ECW/BCM, LTI, BCMI, LTM/weight and BCM/weight.**


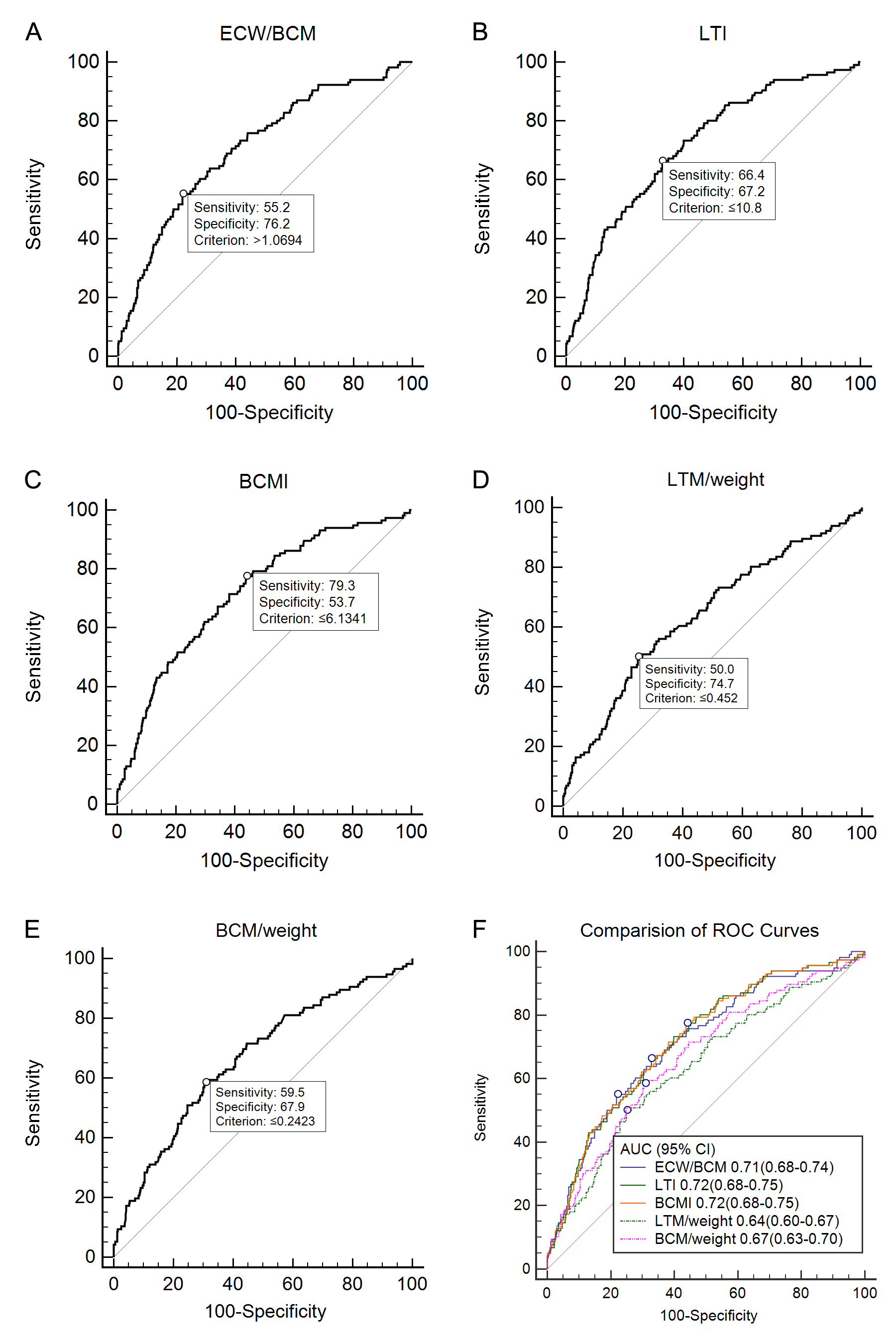


**Note:** There were not significantly differences of predictive values for 2-year all-cause mortality in three parameters ECW/BCM, LTI and BCMI. The C statistics of ECW/BCM, LTI and BCMI for predicting 2-year all-cause mortality were significantly larger than that of LTM/weight and BCM/weight.

**Abbreviations:** BCM, body cell mass; BCMI, body cell mass index; ECW/BCM, the ratio of extracellular water and body cell mass; LTI, lean tissue index; LTM, lean tissue mass.

**Table S1. Matrix of Spearman correlation coefficients between measured parameters (N = 704).**

|  | **MAP** | **OH** | **OH/ECW** | **ECW/BCM** | **ECW/ICW** | **BMI** | **FTI** | **ATM/w** | **LTI** | **LTM/w** | **BCMI** | **BCM/w** |
| --- | --- | --- | --- | --- | --- | --- | --- | --- | --- | --- | --- | --- |
| **MAP** | 1.000 | 0.196 | 0.187 | -0.019 | 0.075 | 0.055 | -0.072 | -0.136 | 0.159 | 0.088 | 0.158 | 0.109 |
|  |  | <.0001 | <.0001 | 0.618 | 0.048 | 0.142 | 0.055 | 0.000 | <.0001 | 0.019 | <.0001 | 0.004 |
| **OH** | 0.196 | 1.000 | 0.987 | 0.430 | 0.743 | 0.023 | -0.164 | -0.240 | 0.022 | -0.021 | 0.009 | -0.025 |
|  | <.0001 |  | <.0001 | <.0001 | <.0001 | 0.541 | <.0001 | <.0001 | 0.563 | 0.578 | 0.806 | 0.509 |
| **OH/ECW** | 0.187 | 0.987 | 1.000 | 0.461 | 0.762 | -0.060 | -0.193 | -0.236 | -0.053 | -0.029 | -0.064 | -0.048 |
|  | <.0001 | <.0001 |  | <.0001 | <.0001 | 0.111 | <.0001 | <.0001 | 0.156 | 0.444 | 0.092 | 0.204 |
| **ECW/BCM** | -0.019 | 0.430 | 0.461 | 1.000 | 0.900 | 0.282 | 0.596 | 0.670 | -0.781 | -0.849 | -0.788 | -0.879 |
|  | 0.618 | <.0001 | <.0001 |  | <.0001 | <.0001 | <.0001 | <.0001 | <.0001 | <.0001 | <.0001 | <.0001 |
| **ECW/ICW** | 0.075 | 0.743 | 0.762 | 0.900 | 1.000 | 0.244 | 0.352 | 0.353 | -0.514 | -0.602 | -0.522 | -0.616 |
|  | 0.048 | <.0001 | <.0001 | <.0001 |  | <.0001 | <.0001 | <.0001 | <.0001 | <.0001 | <.0001 | <.0001 |
| **BMI** | 0.055 | 0.023 | -0.060 | 0.282 | 0.244 | 1.000 | 0.768 | 0.525 | 0.109 | -0.534 | 0.106 | -0.400 |
|  | 0.142 | 0.541 | 0.111 | <.0001 | <.0001 |  | <.0001 | <.0001 | 0.004 | <.0001 | 0.005 | <.0001 |
| **FTI** | -0.072 | -0.164 | -0.193 | 0.596 | 0.352 | 0.768 | 1.000 | 0.939 | -0.469 | -0.908 | -0.469 | -0.838 |
|  | 0.055 | <.0001 | <.0001 | <.0001 | <.0001 | <.0001 |  | <.0001 | <.0001 | <.0001 | <.0001 | <.0001 |
| **ATM/w** | -0.136 | -0.240 | -0.236 | 0.670 | 0.353 | 0.525 | 0.939 | 1.000 | -0.706 | -0.952 | -0.704 | -0.933 |
|  | 0.000 | <.0001 | <.0001 | <.0001 | <.0001 | <.0001 | <.0001 |  | <.0001 | <.0001 | <.0001 | <.0001 |
| **LTI** | 0.159 | 0.022 | -0.053 | **-**0.781 | -0.514 | 0.109 | -0.469 | -0.706 | 1.000 | 0.748 | 0.999 | 0.841 |
|  | <.0001 | 0.563 | 0.156 | <.0001 | <.0001 | 0.004 | <.0001 | <.0001 |  | <.0001 | <.0001 | <.0001 |
| **LTM/w** | 0.088 | -0.021 | -0.029 | -0.849 | -0.602 | -0.534 | -0.908 | -0.952 | 0.748 | 1.000 | 0.749 | 0.985 |
|  | 0.019 | 0.578 | 0.444 | <.0001 | <.0001 | <.0001 | <.0001 | <.0001 | <.0001 |  | <.0001 | <.0001 |
| **BCMI** | 0.158 | 0.009 | -0.064 | -0.788 | -0.522 | 0.106 | -0.469 | -0.704 | 0.999 | 0.749 | 1.000 | 0.844 |
|  | <.0001 | 0.806 | 0.092 | <.0001 | <.0001 | 0.005 | <.0001 | <.0001 | <.0001 | <.0001 |  | <.0001 |
| **BCM/w** | 0.109 | -0.025 | -0.048 | -0.879 | -0.616 | -0.400 | -0.838 | -0.933 | 0.841 | 0.985 | 0.844 | 1.000 |
|  | 0.004 | 0.509 | 0.204 | <.0001 | <.0001 | <.0001 | <.0001 | <.0001 | <.0001 | <.0001 | <.0001 |  |

**Abbreviations:** ATM, adipose tissue mass; BCM, body cell mass; BCMI, body cell mass index; BMI, body mass index; ECW, extracellular water; ECW/BCM, the ratio of extracellular water and body cell mass; ECW/ICW, the ratio of extracellular water and intracellular water; FTI, fat tissue index; LTI, lean tissue index; LTM, lean tissue mass; MAP, mean arterial pressure; OH, overhydration; OH/ECW, the ratio of overhydration and extracellular water.

**Table S2. Univariate COX regression models (N=704).**

| **Variables** | **HR (95%Cl)** | **P value** |
| --- | --- | --- |
| **Demographic data** |  |  |
| Age (per 1-year increment) | 1.05(1.04-1.06) | <0.001* |
| Sex (male *vs* female) | 0.80(0.58-1.09) | 0.155 |
| Height (per 1-cm increment) | 0.98(0.96-1.00) | 0.045* |
| Weight (per 1-Kg increment) | 0.98(0.97-1.00) | 0.014* |
| SBP (per 1 mmHg increment) | 1.00(0.99-1.01) | 0.617 |
| DBP (per 1 mmHg increment) | 0.97(0.96-0.98) | <0.001* |
| MAP (per 1 mmHg increment) | 0.99(0.98-1.00) | 0.008 |
| **Comorbidity (n, %)** |  |  |
| Hypertension (Yes *vs* No) | 1.12(0.75-1.67) | 0.585 |
| Diabetes (Yes *vs* No) | 2.94(2.14-4.02) | <0.001* |
| Infection (Yes *vs* No) | 2.14(1.56-2.93) | <0.001* |
| Smoking history (Yes *vs* No) | 1.21(0.81-1.82) | 0.346 |
| Modified CCI (per 1 score increment) | 1.45(1.30-1.62) | <0.001* |
| **Reasons for admission** |  |  |
| Vascular access | 1.20(0.86-1.67) | 0.288 |
| Infection | 2.58(1.80-3.71) | <0.001* |
| Cardiovascular diseases | 2.04(1.27-3.30) | 0.004* |
| Receiving parathyroidectomy | 0.18(0.11-0.29) | <0.001* |
| Others | 1.75(1.18-2.60) | 0.005* |
| **Dialysis data** |  |  |
| Incident dialysis (Yes vs No) | 1.22(0.88-1.71) | 0.237 |
| Dialysis vintage (per SD increment) | 0.76(0.63-0.90) | 0.002* |
| DVC *vs* no-DVC | 1.43(1.05-1.96) | 0.025* |
| **Laboratory data** |  |  |
| Hemoglobin (per 1 increment) | 0.99(0.99-1.00) | 0.062 |
| Albumin (per 1 g/L increment) | 0.93(0.91-0.96) | <0.001* |
| TC (per 1 mmol/L increment) | 0.97(0.86-1.10) | 0.620 |
| TG (per 1 mmol/L increment) | 1.07(0.99-1.14) | 0.072 |
| HDL-C (per 1 mmol/L increment) | 0.44(0.26-0.74) | 0.002* |
| LDL (per 1 mmol/L increment) | 0.98(0.83-1.16) | 0.826 |
| Adjusted Calcium (per 1 mmol/L) | 0.50(0.28-0.88) | 0.016* |
| Phosphorus (per 1 mmol/L) | 0.34(0.25-0.46) | <0.001* |
| Intact parathyroid hormone (per 100 pg/mL) | 0.92(0.89-0.94) | <0.001* |

**Note:** *P<0.05.

**Abbreviations:** modified CCI, modified Charlson comorbidity index (excluding assessment of diabetes); SBP, systolic blood pressure; DBP, diastolic blood pressure; MAP, mean arterial pressure; HDL-C, high density lipoprotein cholesterol, LDL-C, low density lipoprotein cholesterol.

**Table S3. Multivariate COX regression analysis of risk factors for death using traditional parameters except parameters in body composition.**

| **Variables** | **HR (95%Cl)** | **P value** |
| --- | --- | --- |
| Age (per 1-year increment) | 1.04(1.02-1.05) | <0.001 |
| Weight (per 1-Kg increment) | 0.97(0.95-0.98) | <0.001 |
| Diabetes (Yes *vs* No) | 1.63(1.15-2.30) | 0.006 |
| Modified CCI (per 1 score increment) | 1.20(1.05-1.36) | <0.001 |
| Albumin (per 1 g/L increment) | 0.95(0.92-0.98) | <0.001 |
| HDL-C (per 1 mmol/L increment) | 0.45(0.27-0.76) | 0.003 |
| Incident dialysis (Yes *vs* No) | 0.55(0.37-0.80) | 0.002 |
| Admission due to receiving parathyroidectomy | 0.31(0.18-0.54) | <0.001 |

**Note:** All the variables that with P <0.10 were included in the multivariate cox regression with stepwise selection.

**Abbreviations:** modified CCI, Charlson comorbidity index (excluding assessment of diabetes); HDL-C, high density lipoprotein cholesterol.

**Table S4. Summary of restricted cubic splines for the association between anthropometric parameters in fluid volume and mortality.**

| Variables | Linearity Tests | | RCS Shape | Restricted cubic spline |
| --- | --- | --- | --- | --- |
|  | χ^2^ | P value |  |  |
| MAP | 11.80 | 0.008 | S-shape | 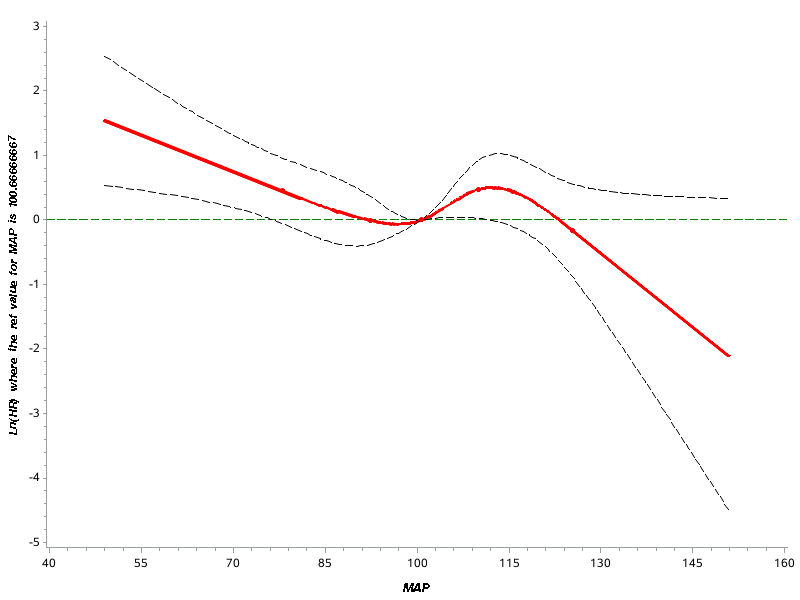 |
| OH | 4.85 | 0.182 | Linear | 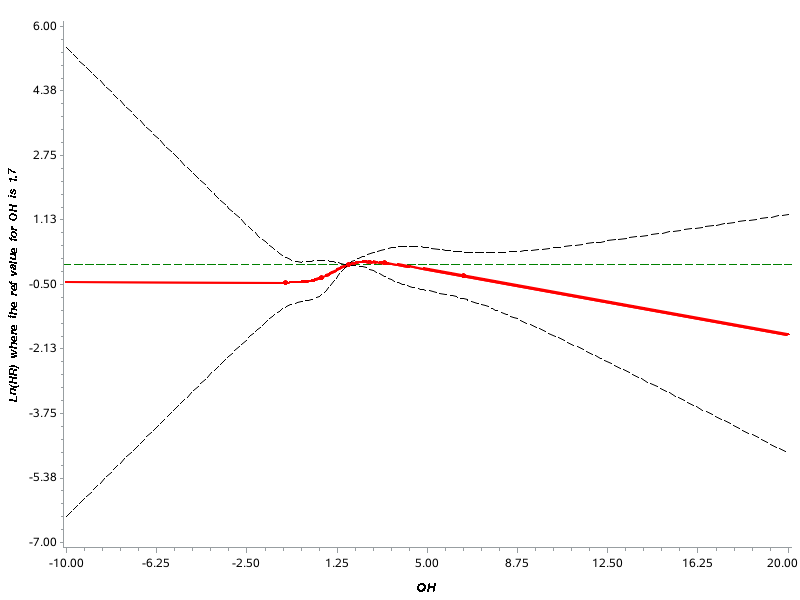 |
| OH/ECW | 3.20 | 0.362 | Linear | 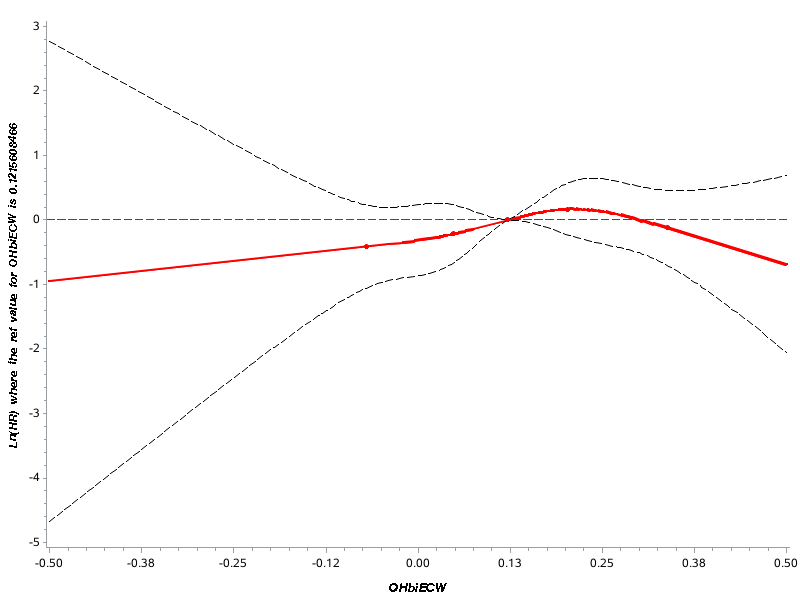 |
| ECW/BCM | 0.92 | 0.820 | Linear | 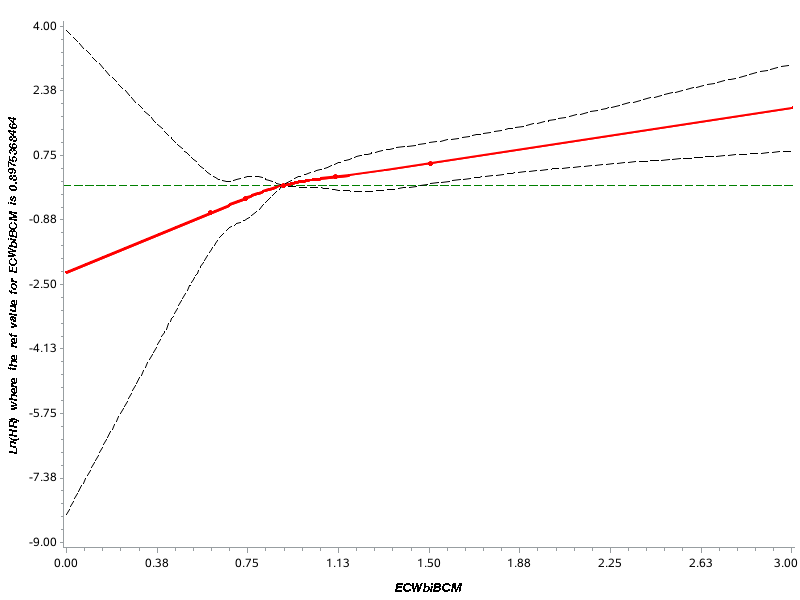 |
| ECW/ICW | 7.97 | 0.047 | S-shape | 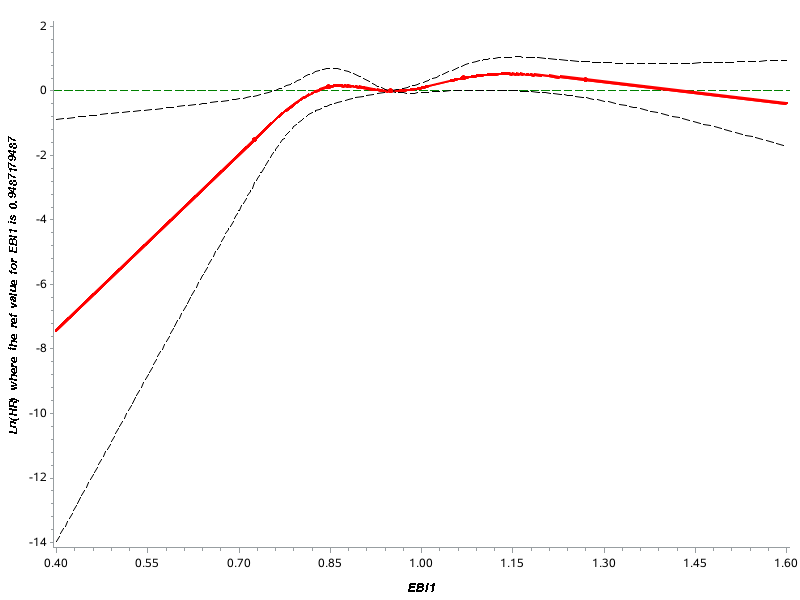 |

**Note:** The restricted cubic splines were adjusted by age, sex, weight, diabetes, modified Charlson comorbidity index, serum albumin concentrations, HDL-C, incident dialysis, smoking status, hypertension, mean blood pressure, dialysis vintage, using deep vein catheter, hemoglobin, total triglycerides, adjusted calcium, phosphorus and iPTH and five admission reasons. Five knots were chosen at 5th, 25th, 50th, 75th, 95th percentiles.

**Abbreviations:** MAP, mean arterial pressure; OH, overhydration; OH/ECW, the ratio of overhydration and extracellular water; ECW/BCM, the ratio of extracellular water and body cell mass; ECW/ICW, the ratio of extracellular water and intracellular water; RCS, restricted cubic spline.

**Table S5. Summary of restricted cubic splines for the association between anthropometric parameters in nutritional status and mortality.**

| Variables | Linearity Tests | | RCS Shape | Restricted cubic spline |
| --- | --- | --- | --- | --- |
|  | χ^2^ | P value |  |  |
| BMI | 6.27 | 0.099 | Linear | 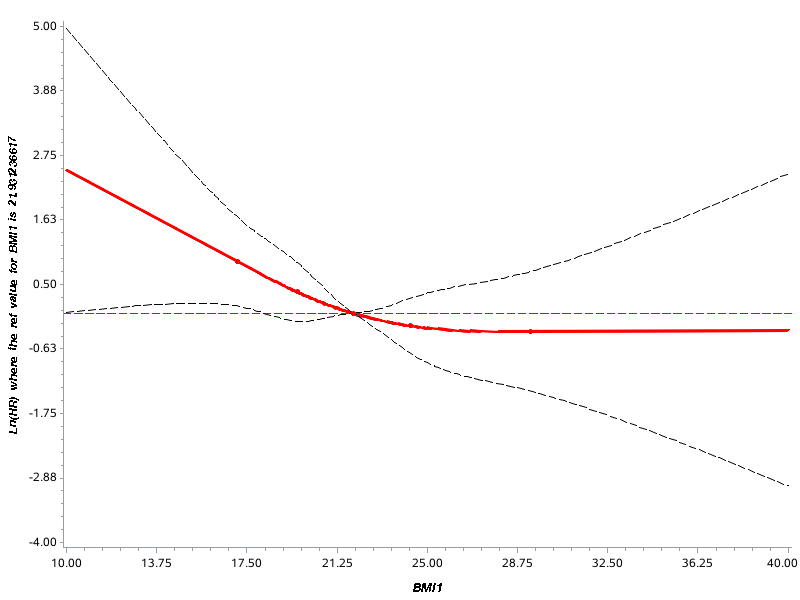 |
| FTI | 10.60 | 0.014 | U-shape | 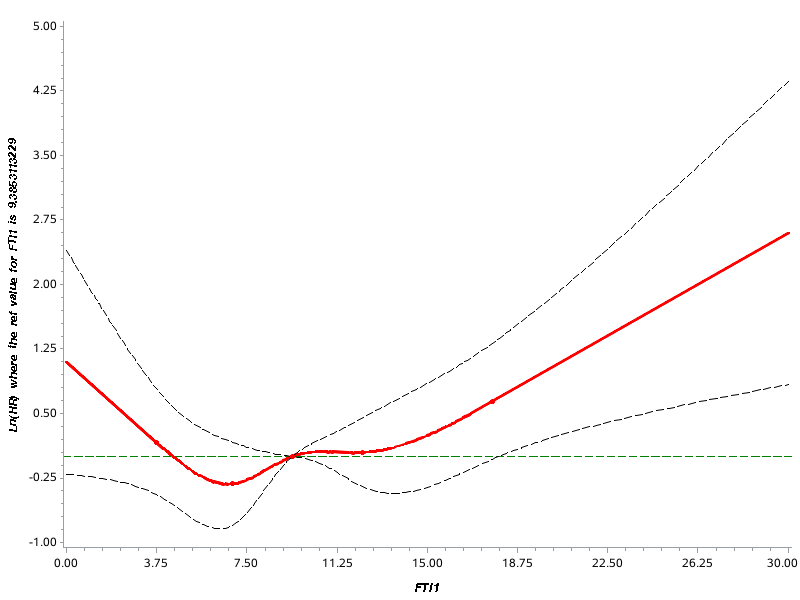 |
| ATM/weight | 8.42 | 0.038 | U-shape | 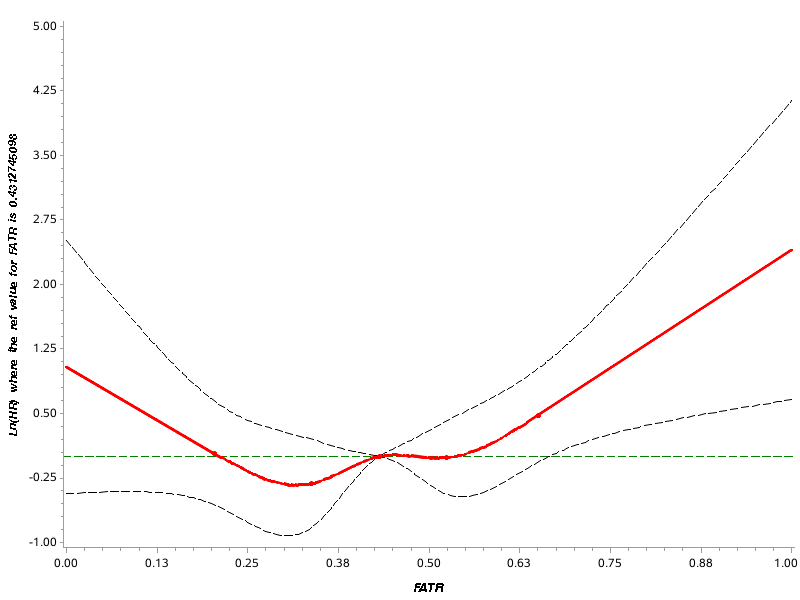 |
| LTI | 2.89 | 0.408 | Linear | 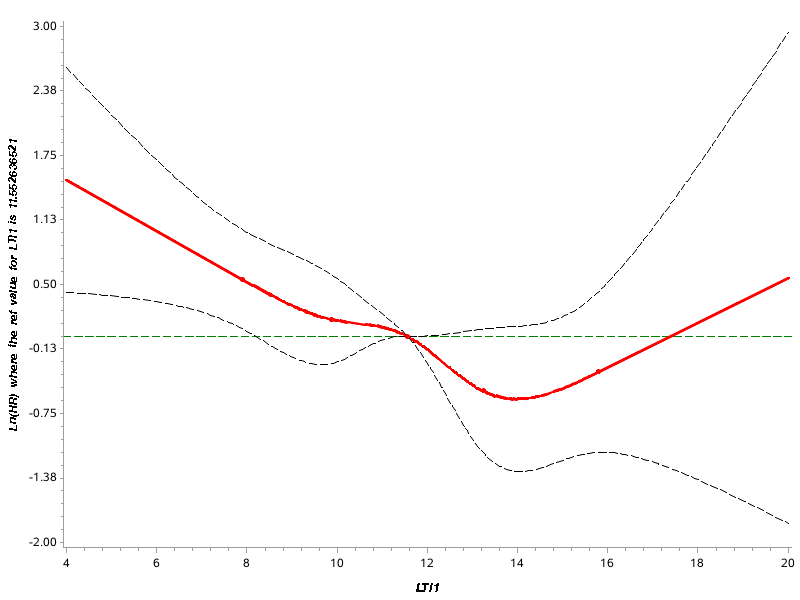 |
| LTM/weight | 6.39 | 0.094 | Linear | 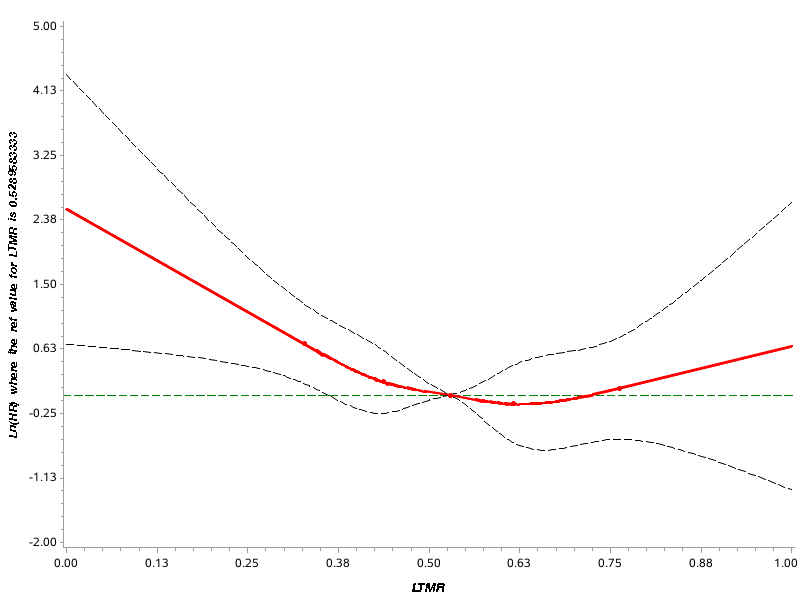 |
| BCMI | 3.25 | 0.355 | Linear | 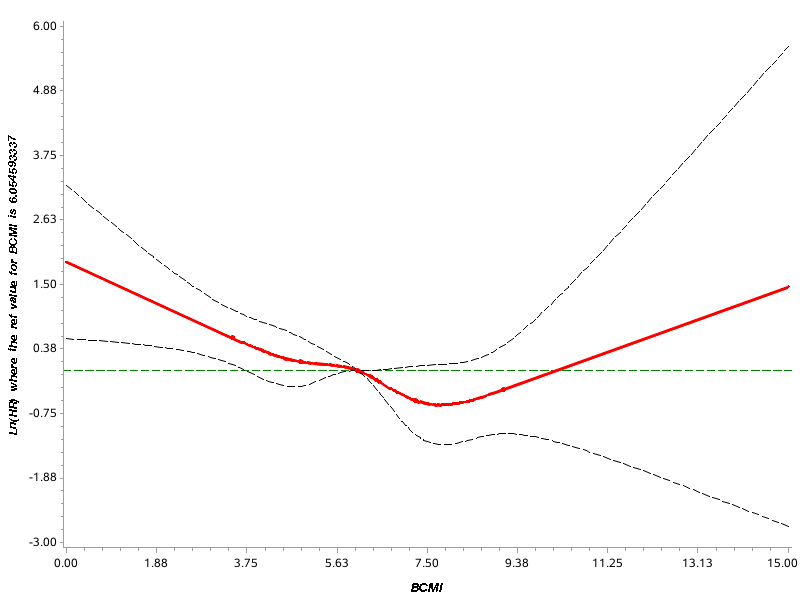 |
| BCM/weight | 7.13 | 0.068 | Linear | 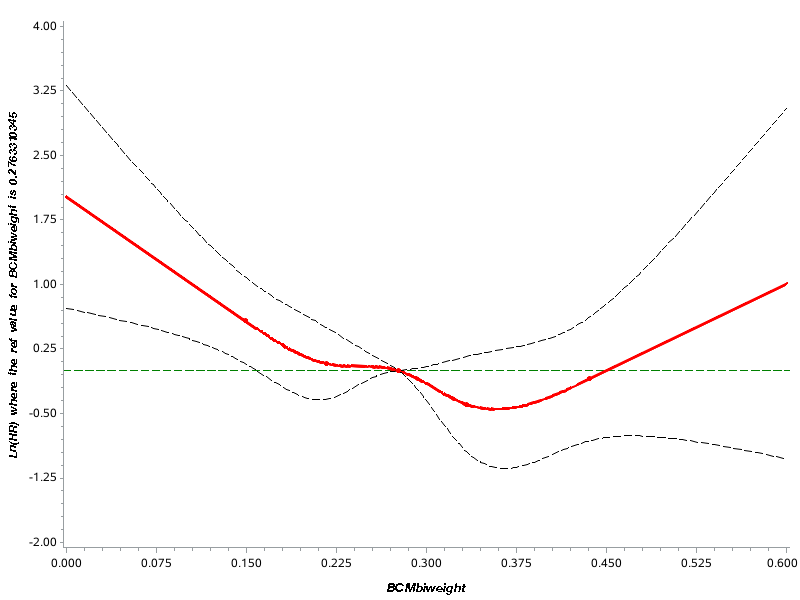 |

**Note:** The restricted cubic splines were adjusted by age, sex, weight, diabetes, modified Charlson comorbidity index, serum albumin concentrations, HDL-C, incident dialysis, smoking status, hypertension, mean blood pressure, dialysis vintage, using deep vein catheter, hemoglobin, total triglycerides, adjusted calcium, phosphorus and iPTH and five admission reasons. Five knots were chosen at 5th, 25th, 50th, 75th, 95th percentiles.

**Abbreviations:** BMI, body mass index; FTI, fat tissue index; ATM, adipose tissue mass; LTI, lean tissue index; LTM, lean tissue mass; BCMI, body cell mass index; BCM, body cell mass; RCS, restricted cubic spline.
